# Navigating TIPS in the era of ageing and metabolic comorbidity: a contemporary narrative review of risk, resilience, and outcomes

**DOI:** 10.1093/gastro/goag103

**Published:** 2026-09-25

**Authors:** Davide Roccarina, Dario Saltini, Chiara Mangini, Antonio Piscopo, Alessio Mastro, Alessandra Barbera, Andrea Salomè Velasco Mayorga, Alessandra Avossa, Lucia Ragozzino, Margherita Falcini, Martina Rosi, Alfredo Vozza, Gianmarco Falcone, Fabrizio Fanelli, Fabio Marra, Sara Montagnese, Filippo Schepis, Francesco Vizzutti

**Affiliations:** Department of Experimental and Clinical Medicine, University of Florence, Florence, Italy; Severe Liver Diseases Departmental Unit (M.E.C.), AOU Policlinico of Modena, CHIMOMO Department University of Modena and Reggio Emilia, Modena, Italy; Department of Medicine (DIMED), University of Padova, Padova, Italy; Severe Liver Diseases Departmental Unit (M.E.C.), AOU Policlinico of Modena, CHIMOMO Department University of Modena and Reggio Emilia, Modena, Italy; Gastroenterology Unit, University Hospital of Modena “Policlinico”, University of Modena and Reggio Emilia, Modena, Italy; Department of Experimental and Clinical Medicine, University of Florence, Florence, Italy; Department of Medicine (DIMED), University of Padova, Padova, Italy; Severe Liver Diseases Departmental Unit (M.E.C.), AOU Policlinico of Modena, CHIMOMO Department University of Modena and Reggio Emilia, Modena, Italy; Gastroenterology Unit, University Hospital of Modena “Policlinico”, University of Modena and Reggio Emilia, Modena, Italy; Department of Experimental and Clinical Medicine, University of Florence, Florence, Italy; Department of Experimental and Clinical Medicine, University of Florence, Florence, Italy; Department of Experimental and Clinical Medicine, University of Florence, Florence, Italy; Department of Experimental and Clinical Medicine, University of Florence, Florence, Italy; Department of Experimental and Clinical Medicine, University of Florence, Florence, Italy; Department of Radiology, Interventional Radiology Unit, Careggi University Hospital, Florence, Italy; Department of Radiology, Interventional Radiology Unit, Careggi University Hospital, Florence, Italy; Department of Experimental and Clinical Medicine, University of Florence, Florence, Italy; Department of Medicine (DIMED), University of Padova, Padova, Italy; Severe Liver Diseases Departmental Unit (M.E.C.), AOU Policlinico of Modena, CHIMOMO Department University of Modena and Reggio Emilia, Modena, Italy; EpatoGastro Lab—RBA-Labs CHIMOMO Department, University of Modena and Reggio Emilia, Modena, Italy; Department of Experimental and Clinical Medicine, University of Florence, Florence, Italy; Portal Hypertension Departmental Unit, Dipartimento Oncologico e di chirurgia ad indirizzo robotico, Azienda Ospedaliero Universitaria Careggi, Florence, Italy

**Keywords:** portal hypertension, under-dilated endoprosthesis, hepatic encephalopathy, older adults, cirrhosis

## Abstract

The ageing of the cirrhotic population, together with the epidemiological shift toward metabolic dysfunction-associated steatohepatitis, is redefining the profile of patients referred for transjugular intrahepatic portosystemic shunt (TIPS). Although TIPS remains a cornerstone in the management of complications of portal hypertension, its abrupt hemodynamic and metabolic effects challenge the physiological reserve of multiple organ systems. Age-related cardiovascular remodeling, cirrhotic cardiomyopathy, impaired renal function and autoregulation, and reduced neurocognitive reserve interact with the hyperdynamic circulatory state of cirrhosis increasing the susceptibility to heart failure, acute kidney injury (AKI), and hepatic encephalopathy following TIPS. This vulnerability is further amplified by metabolic comorbidities, obesity, sarcopenia, frailty, and age-related alterations of the gut microbiota and gut–liver axis, which collectively promote systemic inflammation, immune dysregulation, endothelial dysfunction, and reduced metabolic adaptability. Rather than independently, these factors shape an interconnected heart–liver–kidney–brain network in which dysfunction in one domain may rapidly propagate to others. This review summarizes how ageing influences the cardiovascular, renal, neurological, and metabolic response to TIPS, critically appraises current strategies for pre-procedural risk assessment, and discusses approaches to improve patient selection and peri-procedural management. Because chronological age alone inadequately reflects procedural risk and clinical outcomes, TIPS evaluation should incorporate a structured assessment of biological ageing, including cardiovascular and renal reserve, neurocognitive vulnerability, sarcopenia, frailty, body-composition phenotype, and metabolic burden. Integrating these domains into pre-procedural assessment may improve patient selection, inform shunt sizing, and ultimately optimize outcomes in this rapidly evolving patient population.

## Introduction

Transjugular intrahepatic portosystemic shunt (TIPS) has undergone a profound evolution since its introduction in the late 1980s [1]. Once considered a high-risk rescue intervention, TIPS is now an established therapeutic option for severe complications of portal hypertension (PH), supported by advances in radiological techniques, stent technology, timing of intervention, patient selection, and peri-procedural optimization [2–6]. As indications have expanded and survival of patients with cirrhosis has improved, the demographic profile of TIPS candidates has also changed, with an increasing proportion of older adults being considered for portal decompression.

This shift reflects broader epidemiological changes in chronic liver disease. Population ageing, together with the rising prevalence of metabolic and renal comorbidities, including metabolic dysfunction-associated steatohepatitis (MASH), hypertension, diabetes, obesity, and chronic kidney disease (CKD), has generated a growing cohort of older patients with decompensated advanced chronic liver disease [7–14]. Compared with younger individuals, older adults with cirrhosis more frequently present with multimorbidity, polypharmacy, frailty, sarcopenia, cognitive impairment, and reduced functional reserve, all of which may complicate the assessment of TIPS candidacy and influence post-procedural outcomes [15–20]. Furthermore, current prognostic models remain limited, as they do not fully capture the multidimensional complexity of contemporary patients with cirrhosis [21, 22].

The physiological stress imposed by TIPS is substantial [23] and challenges cardiovascular reserve, renal autoregulation, and cerebral metabolic stability, domains that often exhibit reduced resilience in older adults [24]. It is therefore unsurprising that age has emerged as a major predictor of post-TIPS complications, including heart failure, acute and acute-on-chronic kidney injury, and hepatic encephalopathy (HE) [25–27].

Despite the increasing clinical relevance of this population, the available evidence remains limited, as older adults are underrepresented in randomized trials and prospective cohorts. Moreover, current data largely derive from studies (Table 1) which are limited by their retrospective design and incomplete reporting of comorbidities [25, 28, 29], cirrhosis etiology [29], TIPS indication [29], device characteristics, hemodynamic response, follow-up, HE severity [30], and transplant eligibility, making optimal risk stratification, patient selection, and peri-procedural management uncertain. Nevertheless, when reported, MASH is more prevalent among older adults than younger patients [28, 30, 31], and refractory ascites more commonly represents the indication for TIPS in older cohorts. As a result, these patients are often more clinically compromised at baseline, with more advanced hepatic, renal, and cardiovascular dysfunction, as well as a higher prevalence of frailty and sarcopenia-related conditions [28]. These findings suggest that older TIPS candidates may represent a particularly vulnerable subgroup, characterized by a higher burden of metabolic liver disease, portal hypertensive decompensation, and hepatic, renal, cardiovascular, and frailty-related impairment.

**Table 1:** Study characteristics for patients aged < 70 vs ≥70 years.^a^

Ref.	Design/Device	Pts distribution	Group imbalances				Outcome			
			Cirrhosis etiology	Functional scores	TIPS indication	Comorbidity	PH complications control	HE	Hospital access	Mortality
[25]	Retrospective/NA	Age ≥70 years: 16/225	Comparable between groups	Comparable between groups	Higher prevalence of RA in younger patients	NA	ND	Comparable in the short term, higher at 1 year in older adults	ND	Comparable in the short term, higher at 1 year in older adults
[28]	Retrospective/stent grafts	Age ≥70 years: 51/451 (after matching 50/50)	Higher prevalence of MASH and higher creatinine levels in older adults	Comparable between groups	Higher prevalence of RA in older adults	NA	ND	Higher in older adults	Higher in older adults	ND at 30 days
[30]	Retrospective/stent grafts	Age ≥70 years: 65/539	Higher prevalence of MASH and cryptogenic cirrhosis in older adults	Comparable between groups	Comparable between groups	Higher creatinine levels in older adults, with no differences according to the modified Charlson comorbidity Index	NA	NA	NA	Higher at 90 days in older adults
[31]	Retrospective/stent grafts	Age ≥70 years: 106/593	Higher prevalence of MASH in older adults and of AUD in younger adults	Comparable between groups	Comparable between groups	T2DM, hypertension and CAD in older adults	NA	ND	ND—higher Multiple TIPS-associated readmission within 12 months in older adults	ND at 90 days and 1 year
[29]	Retrospective/stent-grafts	Age ≥70 years: 35/136	NA	Lower MELD score in older adults	NA	NA	NA	ND at 30 days	ND at 30 days	Higher at 30 and 90 days in older adults
[34]	Retrospective analysis of pts prospectively collected/stent grafts	Age ≥70 years: 99/411 and 76/415 in derivation and validation cohorts, respectively	Higher prevalence of AUD in younger patients	Comparable between groups	Comparable between groups	Comparable between groups	ND	ND	NA	ND in TFS was observed up to 5 years

a AUD, alcohol use disorder; CAD, coronary artery disease; MASH, metabolic dysfunction-associated steatohepatitis; MELD, model for end-stage liver disease; NA, not available; ND, no difference; RA, refractory ascites; T2DM, type 2 diabetes mellitus; TFS, transplant-free survival.

Interestingly, despite using a 65-year age cut-off, two studies that included a substantial proportion of older adults support the feasibility and safety of TIPS in this population. In patients with refractory ascites (RA), Stockhoff *et al.* [32] found that age independently predicted 90-day mortality, but not 28-day or 1-year survival, concluding that TIPS is feasible in carefully selected older patients. Similarly, Bisht *et al.* [33] reported no significant age-related differences in HE, post-TIPS complications, or mortality, supporting TIPS as an equally safe option for older adults.

In our previous study [34], conducted in a large cohort of carefully selected older patients undergoing TIPS for RA or secondary prophylaxis of variceal bleeding, an externally validated model incorporating creatinine and sodium was able to predict liver-related mortality after TIPS. A distinctive feature of this cohort was the systematic use of stent-graft under-dilation, which was more frequently applied in older adults and may have helped mitigate the impact of age-related multisystem vulnerability. Notably, transplant-free survival did not differ significantly between younger and older patients for more than 5 years after TIPS. However, these findings should be interpreted with caution, as transplant-free survival is an imperfect endpoint in this setting. Yet, also our experience did not fully characterize the frailty-related and multisystem determinants of post-TIPS outcomes.

After summarizing the current knowledge on the use of TIPS in older adults, this review will focus on the cardiovascular, renal, metabolic, and neurocognitive domains most affected by ageing. It aims to provide a multidisciplinary framework for individualized assessment of TIPS candidacy and peri-procedural care, while highlighting the need for dedicated prospective studies comparing TIPS with standard-of-care management and incorporating health-related quality of life outcomes.

## Physiological and clinical characteristics of ageing relevant to TIPS

**Figure 1:**
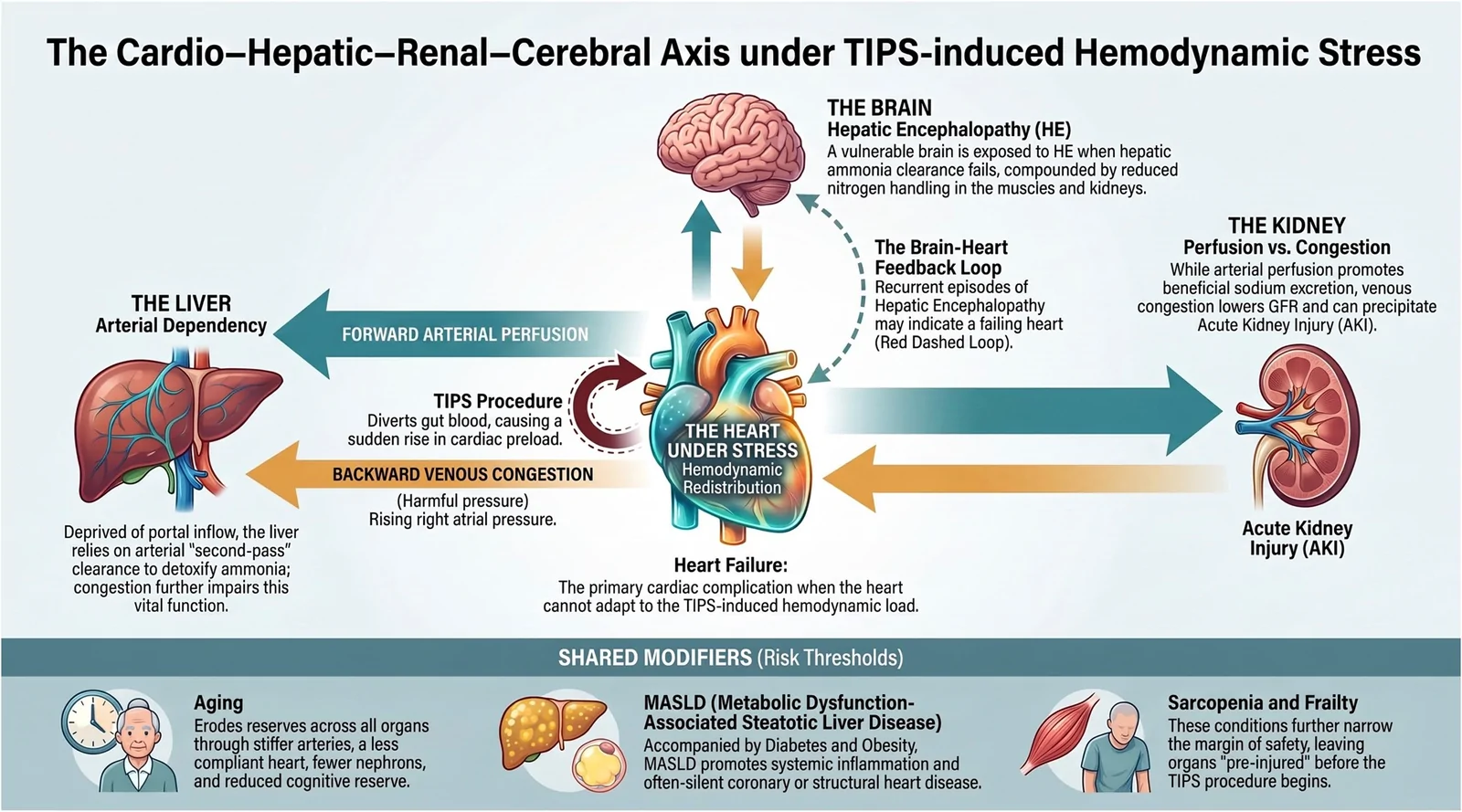
After a TIPS, hemodynamic changes extend beyond the liver, affecting the heart, kidney, and brain. TIPS diverts splanchnic blood into the systemic circulation, increasing cardiac preload while reducing portal inflow, making the liver dependent on hepatic arterial perfusion, and thus cardiac output, for viability and residual ammonia detoxification. The heart influences each organ through two opposing mechanisms: forward arterial perfusion (teal arrows), which supports function, and backward venous congestion (amber arrows), which develops when the heart cannot accommodate the increased preload, leading to elevated right atrial pressure and post-capillary pulmonary hypertension. In the liver, arterial perfusion sustains residual (second pass) ammonia clearance, whereas congestion worsens hepatic perfusion and function. In the kidney, improved perfusion enhances sodium excretion, while congestion reduces GFR and may cause AKI. Reduced hepatic ammonia clearance, together with impaired muscle and renal nitrogen handling, increases the risk of HE. The dashed red loop indicates the emerging hypothesis that recurrent post-TIPS HE may reflect underlying cardiac dysfunction. Indeed, the marked increase in preload following TIPS may unmask limited cardiac reserve, with subsequent systemic and hepatic venous congestion potentially contributing to impaired hepatic clearance and recurrent HE. This hypothesis is biologically plausible but remains insufficiently investigated and warrants prospective validation, particularly in older patients. An additional, yet insufficiently explored, mechanism may involve impaired cerebral perfusion in the context of an age-related reduction in cerebrovascular reserve and potentially impaired cerebral autoregulation. Reduced cerebral blood flow may increase cerebral vulnerability to hyperammonaemia and thereby contribute to recurrent or persistent post-TIPS HE. Each colored box identifies the principal post-TIPS complication of each organ, while the lower band highlights shared modifiers. Ageing reduces cardiac, hepatic, renal and cognitive reserve, lowering tolerance to hemodynamic stress. MASLD (metabolic dysfunction-associated steatotic liver disease), often associated with obesity and type 2 diabetes, adds systemic inflammation, endothelial dysfunction, occult cardiovascular disease and CKD, leaving each organ more vulnerable before TIPS. Sarcopenia and frailty further reduce physiological reserve. Solid arrows indicate established relationships, whereas the dashed arrow represents an emerging/unpublished link. AKI, acute kidney injury; CKD, chronic kidney diseas; GFR, glomerular filtration rate; HE, hepatic encephalopathy; MASLD, metabolic dysfunction-associated steatotic liver disease; N, nitrogen; TIPS, transjugular intrahepatic portosystemic shunt.

The hemodynamic consequences of TIPS do not respect organ boundaries. By diverting portal flow into the systemic circulation, TIPS imposes an acute preload challenge on the heart, modifies hepatic perfusion, exposes the kidney to competing forces of improved forward flow and venous congestion, and increases systemic exposure to gut-derived neurotoxins. In older and metabolically compromised patients, reduced reserve across these interconnected domains may transform portal decompression into a multisystem stress test. This framework underpins the following sections, which examine neurocognitive, cardiovascular, renal, metabolic, frailty-related, and microbiota-mediated vulnerability (Fig. 1).

### Neurocognitive vulnerability

Age is a well-established risk factor for post-TIPS HE, and has consistently been identified as a significant predictor of post-TIPS HE, along with other variables, such as the Child–Pugh score, a larger-calibre stent, a history of overt HE (OHE) [23, 25, 35, 36], the presence of covert HE (CHE) [37–39], and a larger difference between pre- and post-TIPS ammonia levels [40].

More specifically, older age (≥ 70 years) has been associated with a higher incidence of post-TIPS OHE, HE-related hospital readmissions, and increased 30- and 90-day mortality rates following TIPS placement [25, 27, 28, 33, 41].

Several mechanisms increase the susceptibility to post-TIPS HE in older patients, including age-related structural and functional cerebral alterations, which make the central nervous system more vulnerable to the effects of neurotoxins, such as ammonia and pro-inflammatory cytokines, and the systemic alterations, which are frequently observed in patients with age-related comorbidities, including sarcopenia [42], CKD, and cardiovascular diseases [26].

Some of the mechanisms that increase neurocognitive susceptibility in older adults include:

- an amplification of baseline neuroinflammation—particularly mediated by IL-6, IL-1β, and TGF-β, which makes the senescent brain more susceptible to the effects of ammonia and systemic inflammatory cytokines [43];
- the alteration of synaptic plasticity with the upregulation of GABRG1/B1 receptors and the consequent enhancement of inhibitory GABAergic tone [44];
- a reduced cognitive reserve, defined as the brain’s ability to adapt to and compensate for brain insults, while preserving cognitive function [45]. Cognitive reserve is influenced by environmental and lifestyle-related factors, such as educational attainment, intellectual engagement and social activity, and physiologically declines with age [46, 47]. Consequently, older patients exhibit a diminished capacity to compensate for insults, such as hyperammonaemia and the accumulation of neurotoxic substances following TIPS placement, thus becoming more susceptible to OHE [48–50]. Cerebral atrophy, which is also more common in older individuals, has also been associated with poor psychometric performance and electroencephalogram alterations in patients with cirrhosis [51];
- the deterioration of the blood–brain barrier, with increased permeability to circulating neurotoxins [52], thereby amplifying the effects of HE-related alterations in proteins essential for its integrity, such as aquaporin-4 and claudin-5 [53].

Further, older patients are frequently under complex pharmacological regimens, which often include psychoactive drugs, which patients with cirrhosis are known to be sensitive to [54]. Two recent studies have shown that cirrhotic patients on psychoactive medication exhibit the OHE phenotype with lower ammonia levels compared to their counterparts on no psychoactive medication [55], and that patients with cirrhosis and acute encephalopathy without hyperammonaemia are more often on psychoactive drugs [56].

It is also possible that tolerance to hyperammonaemia may decrease with age and over time after TIPS placement, as suggested by patients [local experience, 57] who start developing OHE as they become older in the absence of changes in their liver function [58, 59].

A highly challenging aspect in older adults is the differential diagnosis between HE, especially CHE, and other forms of age-related cognitive impairment. CHE mostly affects the cognitive domains of attention, psychomotor speed, executive function, and working memory [60]. By contrast, other forms of age-related cognitive impairment/dementia are dominated by memory impairment, testing for which can also serve as a differential diagnosis tool [local experience, 57]. It is also possible that the motor aspects of HE [61, 62], up to severe forms, such as hepatic myelopathy, which are almost invariably associated with portosystemic shunts [63–65], may impact older adults with any degree of motor impairment more than their younger counterparts.

Given the above considerations, preventative strategies to reduce the risk of post-TIPS HE may be especially relevant to older adults, and dedicated studies seem worthwhile. Further, therapeutic HE management can be more difficult in older adults. Based on our experience in a dedicated HE clinic within a tertiary referral hepatology center, additional therapies may be considered when standard treatment fails, especially considering that add-on HE management strategies have generally favorable safety profiles [66]. Finally, given the challenges in the diagnosis and differential diagnosis of CHE, as well as potential overlap with cognitive impairment from other etiologies, a comprehensive pre-TIPS semiquantitative evaluation for HE may help identify patients at higher risk and improve patient selection [67].

### Cardiovascular reserve

Ageing induces progressive structural and functional cardiovascular changes, driven largely by elastin degradation and collagen deposition: arterial stiffening, left ventricular hypertrophy, and impaired myocardial relaxation. Coupled with decreased baroreflex sensitivity, blunted β-adrenergic responsiveness, and a lower maximal cardiac output, these alterations produce diastolic dysfunction and curtail the chronotropic and inotropic reserve needed to meet physiological stress and, crucially, the capacity to accommodate an acute volume load [68, 69].

These age-related cardiovascular changes are superimposed on the hyperdynamic circulatory state of decompensated advanced chronic liver disease, characterized by high cardiac output, reduced systemic vascular resistance, marked splanchnic vasodilation, and effective arterial hypovolemia, with consequent neurohormonal activation. Against this background, cirrhotic cardiomyopathy further compromises cardiovascular reserve through latent systolic dysfunction that becomes apparent only under stress, together with diastolic dysfunction and electrophysiological abnormalities, including QT interval prolongation and chronotropic incompetence [70–72]. Diastolic dysfunction is particularly consequential, as a stiff, poorly compliant ventricle is less able to accommodate an acute increase in preload because filling pressures rise steeply even with modest increases in ventricular volume [72].

Assessing true cardiovascular reserve in older patients with decompensated cirrhosis is inherently challenging, as their often-sedentary lifestyle may conceal subclinical cardiac dysfunction. Consequently, cardiovascular reserve frequently remains untested until it is challenged by the abrupt hemodynamic changes induced by TIPS [73, 74]. The epidemiological shift toward MASH further compounds the cardiovascular vulnerability associated with both ageing and the hemodynamic consequences of cirrhosis [5, 75]. Metabolic comorbidities further contribute to systemic inflammation, endothelial dysfunction, and microvascular disease, thereby creating a cardiovascular milieu that is particularly vulnerable to hemodynamic stress [76, 77]. Nevertheless, the optimal pre-procedural cardiovascular assessment of patients with MASH remains a matter of debate [78]. Importantly, this phenotype is strongly associated with silent coronary artery disease, raising the concern that TIPS-induced hemodynamic stress may unmask previously occult ischemia.

TIPS acutely increases venous return by diverting splanchnic blood into the systemic circulation, raising preload, cardiac output, and right-sided filling and pulmonary pressures [79, 80]. Younger patients may absorb this surge, but the aged and metabolically burdened myocardium is prone to inadequate adaptation; notably, in this context, under-dilated TIPS endoprostheses (see Section addressing the unmet needs of older adults undergoing TIPS) have emerged as a strategy to mitigate the risk of cardiovascular decompensation [81]. Pre-existing pulmonary hypertension or right ventricular dysfunction further narrows the margin of tolerance.

Early hemodynamic measurements obtained immediately after TIPS placement should be interpreted with caution. A marked reduction in the portosystemic pressure gradient may, in fact, reflect an increase in central venous pressure secondary to acute right-sided cardiac strain rather than true portal decompression [82, 83]. Indeed, elevated right atrial pressure independently tracks with worse outcomes after TIPS [84] and symptomatic post-TIPS heart failure, frequently arising within weeks to months, has become an increasingly recognized and severe complication [84].

Despite the availability of numerous echocardiographic and biomarker-based predictors, individualized risk stratification remains suboptimal, as current evidence is largely derived from retrospective, heterogeneous studies that provide population-level associations rather than reliable patient-specific risk estimates [24, 34, 83, 85, 86]. Even the prognostic value of diastolic dysfunction remains controversial. While one study has associated it with poorer survival after TIPS [87], another has found no independent predictive value [85], underscoring the limited ability of resting cardiac indices to capture the dynamic cardiovascular response to the acute preload challenge imposed by TIPS.

Accurate prediction of post-TIPS heart failure thus remains elusive [88] and the interplay of ageing, PH, and MASH-related cardiometabolic injury continues to challenge clinical decision-making. In practice, pre-procedural evaluation leans on resting echocardiography, diastolic indices (E/e′, left atrial volume), global longitudinal strain, estimated pulmonary pressures, and right ventricular function, supplemented by natriuretic peptides and, where ischemia is suspected, functional or anatomical coronary assessment [74, 78]. Right heart catheterization should be considered both to identify hemodynamic contraindications that should be weighed against the indication for TIPS, particularly warranting a more cautious approach in patients undergoing TIPS for RA, and to guide post-TIPS therapeutic management. We believe that right heart catheterization should be strongly considered in patients with elevated baseline NT-proBNP levels and inconclusive or equivocal echocardiographic findings, as well as in those with diastolic dysfunction, suspected heart failure with preserved ejection fraction or pulmonary hypertension, and in patients with MASH (Table 2). The predictive value of right heart catheterization in this emerging and increasingly prevalent patient population warrants further investigation, particularly given the expected higher prevalence of ischemic and structural heart disease. It will be important to determine whether a systematic and comprehensive cardiovascular assessment should be incorporated into the pre-procedural evaluation of TIPS candidates, particularly to identify MASH-associated cardiovascular conditions. Each measure, however, captures the heart at rest rather than under the preload it must soon absorb, limiting its negative predictive value [85].

**Table 2:** Clinical, historical, biochemical, and instrumental parameters recommended for assessment in older adults undergoing TIPS to ensure an accurate evaluation of systemic hemodynamic risk.^a^

Parameter/domain	Assessment	High-risk criterion/cut-off
**Cardiovascular history**	History of CVD	Previous cardiovascular disease
**EKG**	EKG	Assessment of electrocardiographic abnormalities
**Biomarkers**	BNP/NT-proBNP	**>300 pg/mL → high risk**
**Functional capacity**	DASI score	**<26 → high risk**
	6MWT	**<250–300 m → high risk**
**Diastolic dysfunction**	≥3 criteria present	**High probability** if ≥3: e′ septal <7 cm/s or lateral <10 cm/s; E/e′ >14; LAVI >34 mL/m²; TRV >2.8 m/s
**RV dysfunction**	Echocardiographic criteria	**High probability** if TAPSE <17 mm; FAC _RV_ <35%; S′ <9.5 cm/s
**LV dysfunction**	Echocardiographic criteria	**High probability** if LVEF <50%; GLS >−18%; MAPSE <10 mm; WMSI >1
**Valvular disease**	Significant valvular disease	Presence of significant valvular disease → **high risk**
**Pulmonary hypertension**	RVSP/TRV	**RVSP >50 mm Hg; TRV >2.8 m/s** (**high risk if >3.8 m/s)**
**Kidney disease**	CKD	Concomitant CKD on **renal replacement therapy → high risk**

RHC parameter	Potentially concerning value for TIPS	Interpretation
**mPAP**	**>35 mm Hg**	Traditionally used threshold for moderate/severe porto-pulmonary hypertension
**PVR**	**>3 WU**	Indicates a significant precapillary component
**RAP**	**>14–15 mm Hg**	Elevated right-sided pressure; a strong marker of congestion and/or risk of cardiac decompensation
**PAWP**	**>15 mm Hg**	Post-capillary pulmonary hypertension; suggests left ventricular dysfunction/HFpEF
**CO/CI**	**Low**	Reduced hemodynamic reserve; increases the risk of cardiac decompensation after an increase in preload
**RAP/PAWP ratio**	**High ratio**	May indicate predominant right-sided cardiac impairment

a See also Fig. 4.

b In the presence of elevated NT-proBNP, an inconclusive or equivocal echocardiogram, diastolic dysfunction, suspected heart failure with preserved ejection fraction, suspected pulmonary hypertension, or MASH, further hemodynamic assessment with right heart catheterization should be considered, potentially including a fluid challenge.

In older adults undergoing TIPS, a comprehensive right heart catheterization assessment should include, at a minimum, RAP, mPAP, PAWP, CO/CI, and PVR, ideally integrated with TAPSE, RV strain, and comprehensive echocardiographic assessment (156). This approach allows the identification of three hemodynamic phenotypes with substantially different clinical implications:.
pre-capillary PH/PoPH → mPAP ↑, PVR ↑, PAWP ≤15 mm Hg → risk primarily related to right ventricular overload and failure;. post-capillary PH/HFpEF → PAWP >15 mm Hg → increased risk of left-sided cardiac decompensation following the increase in preload induced by TIPS;. advanced RV dysfunction/congestion → RAP ↑ ± CI ↓ → likely the most unfavorable hemodynamic profile for TIPS.

It is worth emphasizing that the most recent EASL recommendations (3) specifically highlight the importance of right atrial pressure and right ventricular function in patient selection, as the increase in preload induced by TIPS may precipitate previously subclinical right-sided cardiac dysfunction.

We do not believe that absolute contraindications should be defined *a priori*, except in cases of clinically overt heart failure and/or significant valvular disease, or in the presence of severe abnormalities in the non-invasive or invasive systemic hemodynamic parameters.

Rather, the risk associated with the shunt procedure should be assessed in the context of the indication for TIPS. In patients undergoing rescue or salvage TIPS, the selection criteria should be less stringent than those applied to patients undergoing TIPS for refractory or recurrent ascites, given the different risk-benefit balance of the procedure.

c BNP, B-type natriuretic peptide; DASI, Duke activity status index; CI, cardiac index; CKD, chronic kidney disease; CO, cardiac output; CVD, cardiovascular disease; EKG, electrocardiogram; FAC_RV_, fractional area change of the right ventricle; GLS, global longitudinal strain; LAV1, left atrial volume index; LVEF, left ventricular ejection fraction; MAPSE, mitral annular plane systolic excursion; mPAP, mean pulmonary artery pressure; PAWP, pulmonary artery wedge pressure; PoPH, portopulmonary hypertension; PVR, pulmonary vascular resistance; RAP, right atrial pressure; RHC, right heart right heart catheterization; RSVP, right ventricular systolic pressure; RV, right ventricular; S′, velocity; TAPSE, tricuspid annular plane systolic excursion; TRV, tricuspid regurgitation velocity; WMSI, wall motion score index; WU, Wood units; 6MWT, 6-minute walk test. The values, parameters, and conditions defining a higher risk of systemic hemodynamic compromise are highlighted in bold.

When post-TIPS heart failure occurs, initial management focuses on diuresis and afterload optimization. In refractory cases, shunt reduction or occlusion may be required, although these interventions inevitably restore PH, potentially leading to recurrence of portal hypertensive complications. This asymmetry, in which rescue options are limited and costly, reinforces the primacy of careful candidate selection and conservative shunt sizing over *post hoc* correction [81, 89].

Emerging prospective data underscore how tightly the heart, and the shunted liver are coupled. In a cohort undergoing serial right-heart catheterization and liver stiffness measurement after TIPS, post-capillary pulmonary hypertension at 1 month and a rising, rather than falling, liver stiffness each predicted subsequent cardiac decompensation, with advanced age remaining an independent risk factor [90]. The increase in liver stiffness among patients who developed cardiac decompensation likely reflects the retrograde transmission of elevated right-sided filling pressures to the hepatic sinusoids, providing a structural correlate of the dynamic heart–liver interaction.

### Renal reserve and cardio-renal coupling

Ageing is accompanied by a progressive decline in renal functional reserve, nephron loss, glomerulosclerosis, a falling glomerular filtration rate (GFR), and reduced renal blood flow [91–93]. Impaired autoregulatory capacity and a diminished vasodilatory reserve leave the ageing kidney unable to maintain renal perfusion during hemodynamic stress, increasing its susceptibility to both hypoperfusion and venous congestion while delaying recovery after injury. In parallel, reduced urinary concentrating ability and impaired sodium handling further limit adaptation to the abrupt intravascular volume redistribution induced by TIPS [80, 92, 93].

In cirrhosis, effective arterial underfilling triggers activation of the renin–angiotensin–aldosterone system, the sympathetic nervous system, and antidiuretic hormone release, leading to renal sodium and water retention together with intrarenal vasoconstriction. These mechanisms constitute the pathophysiological substrate for ascites formation and, in its most severe form, hepatorenal syndrome–acute kidney injury (HRS-AKI) [94].

By mobilizing ascites and increasing venous return, TIPS can enhance cardiac output and attenuate neurohormonal activation, thereby improving renal perfusion and sodium excretion. These benefits typically emerge over weeks, in parallel with ascites resolution, and distinguish a sustained renal improvement from the immediate hemodynamic impact of the procedure [80, 95]. However, this favorable effect is critically dependent on cardiac reserve. When the heart is unable to convert the increased preload into forward flow, the kidney is exposed instead to rising central venous pressure, which is transmitted as renal venous congestion. The resulting increase in interstitial and tubular pressures reduces the net filtration gradient and lowers GFR without any compensatory improvement in perfusion. This effect is further exacerbated by elevated intra-abdominal pressure in tense ascites, which is transmitted to the renal veins [80, 94, 96]. The kidney therefore lies at the intersection of two cardiac-dependent forces: forward perfusion and backward venous congestion. HRS-AKI crystallizes this balance. Vasoconstrictors combined with albumin remain the first-line therapy [97], whereas TIPS represents a second-line option in selected patients, capable of relieving renal vasoconstriction and improving renal function in appropriate candidates [96, 98]. However, it may become hazardous when cardiac reserve is insufficient to accommodate the additional hemodynamic load [30]. In older patients, this balance is particularly precarious [95, 98]. Current evidence, largely derived from small series, suggests improvements in renal function and possibly survival in carefully selected individuals with HRS-AKI, although definitive conclusions await larger controlled studies [98].

Conceptually, the kidney after TIPS is exposed to a cardiorenal insult, whereby the same rise in filling pressures that challenges cardiac function is transmitted to the renal circulation. Post-TIPS AKI is itself an adverse prognostic marker. Accordingly, a reduction in urine output or an increase in serum creatinine in the days following the procedure should prompt evaluation of cardiac function and volume status rather than immediate escalation of diuretic therapy. During this dynamic phase, measured or cystatin C-based estimates of GFR, together with serial volume assessment, are more informative than creatinine alone. Indeed, baseline renal function is not adequately captured by serum creatinine, which frequently underestimates the degree of kidney dysfunction in patients with sarcopenia and reduced muscle mass [99]. A comprehensive pre-TIPS renal evaluation is therefore mandatory [3], and preventive measures are paramount to protect the vulnerable ageing kidney: rigorous screening for albuminuria, strict avoidance of nephrotoxic agents and NSAIDs, use of low-osmolar contrast media to minimize the contrast load, and meticulous titration of diuretic therapy after TIPS to prevent intravascular volume depletion during the rapid mobilization of ascites.

A separate consideration applies to patients with advanced CKD. In these individuals, the acute increase in cardiac preload following TIPS cannot be adequately compensated for by an increase in urinary sodium and water excretion. Consequently, they are at increased risk of clinically significant volume overload and pulmonary edema, warranting early nephrology involvement and prompt initiation or intensification of renal replacement therapy when clinically indicated.

Older adults with advanced CKD are also at increased risk of post-TIPS HE. Besides the greater burden of uremic toxins, impaired renal function reduces the kidney’s contribution to systemic ammonia homeostasis, thereby favoring hyperammonemia in the setting of portosystemic shunting. Close post-procedural monitoring and early implementation of preventive strategies for HE are therefore strongly warranted in this high-risk population. Although renal replacement therapy should not be considered a routine treatment for HE, it may provide the additional benefit of ammonia clearance in selected patients with severe hyperammonemia.

### Sarcopenia and frailty

Sarcopenia, defined as the progressive loss of skeletal muscle mass, strength, and physical performance, is one of the most clinically relevant manifestations of metabolic ageing in cirrhosis. Although originally considered an age-related condition, it is now recognized as a frequent complication of chronic liver disease, with reported prevalence ranging from 23% to 70% depending on disease severity, population characteristics, and diagnostic method. Meta-analytic data suggest an overall prevalence of approximately 41%, with higher rates in decompensated disease and in older individuals [100–102].

The pathogenesis of sarcopenia in cirrhosis is multifactorial and reflects the interaction between liver failure, systemic inflammation, malnutrition, hormonal dysregulation, physical inactivity, inadequate protein intake, and altered energy metabolism [103, 104].

Among these mechanisms, hyperammonaemia plays a central role. In cirrhosis, impaired hepatic urea synthesis and portosystemic shunting reduce ammonia clearance, increasing the reliance on skeletal muscle for extrahepatic detoxification through glutamine synthetase. As muscle mass declines, this compensatory pathway becomes progressively less efficient, increasing susceptibility to HE. Conversely, ammonia itself promotes muscle wasting by impairing protein synthesis, inducing mitochondrial dysfunction, activating myostatin signaling, and enhancing autophagy, thereby creating a self-perpetuating cycle of hyperammonaemia, sarcopenia, and HE [105, 106].

This axis is particularly relevant in the setting of TIPS. By increasing portosystemic blood flow and bypassing hepatic first-pass ammonia detoxification, TIPS may increase systemic ammonia exposure. In patients with reduced skeletal muscle reserve, the capacity for extrahepatic ammonia clearance is further impaired, providing a biologically plausible explanation for their increased susceptibility to post-TIPS HE. Accordingly, sarcopenia has emerged as an important predictor of post-TIPS HE and mortality in older adults undergoing TIPS [99]. A recent systematic review and meta-analysis confirmed that sarcopenic patients undergoing TIPS have a significantly higher risk of post-procedural HE and death, supporting the integration of muscle assessment into pre-TIPS risk stratification [107].

Frailty represents a complementary but distinct construct. It is a multidimensional syndrome characterized by reduced strength, mobility, endurance, and physiological reserve, and is increasingly recognized as a determinant of adverse outcomes in older patients with cirrhosis. Assessed using tools, such as the Liver Frailty Index, gait speed, handgrip strength, and functional performance tests, frailty independently predicts mortality, hospitalizations, complications, and poor post-procedural recovery [108]. Its prevalence increases with age and often overlaps with sarcopenia, amplifying vulnerability to acute stressors, including the hemodynamic and metabolic changes induced by TIPS [109, 110].

The relationship between TIPS, sarcopenia, and frailty is complex and may be bidirectional. Frailty and sarcopenia may worsen post-TIPS outcomes, but TIPS itself may also modify nutritional status and body composition when performed for accepted indications. Available evidence suggests that TIPS may be associated with increases in lean body mass, skeletal muscle index, and psoas muscle index, particularly in patients with RA [111, 112].

However, improvements in muscle mass do not consistently translate into better muscle strength, Liver Frailty Index, or physical performance, possibly because of persistent impairment in muscle quality, neuromuscular function, and hyperammonemia-related effects on muscle metabolism [112–114].

Importantly, sarcopenia and frailty are related but not interchangeable. While sarcopenia has been consistently associated with worse post-TIPS outcomes, frailty assessed using current Liver Frailty Index cut-offs does not reliably predict OHE or transplant-free survival after TIPS, suggesting that frailty thresholds may require recalibration in this specific setting [114]. Current guidelines do not support TIPS specifically for the treatment of frailty or sarcopenia but acknowledge that TIPS performed for standard indications may indirectly improve muscle mass [111].

Overall, sarcopenia and frailty identify a subgroup of older cirrhotic patients with reduced metabolic and functional reserve. Reduced muscle mass limits ammonia detoxification, whereas frailty decreases tolerance to procedural stress, hemodynamic shifts, and post-TIPS complications. These data support the integration of objective measures of muscle mass, strength, and functional capacity into pre-TIPS assessment, together with multidisciplinary strategies including nutritional optimization, resistance exercise, prehabilitation, and targeted pharmacologic interventions aimed at improving resilience and reducing post-procedural risk [110, 111, 115]. We believe that future studies should investigate whether the improvement or reversal of sarcopenia and frailty following TIPS translates into a measurable prognostic benefit.

### Diabetes mellitus

Diabetes mellitus (DM) is an important comorbidity in patients undergoing TIPS, as it may influence post-procedural outcomes without representing an absolute contraindication. Its relevance includes the metabolic effects of portosystemic shunting on glucose homeostasis, the increased risk of post-TIPS complications, and patient selection in the MASH era.

TIPS reduces hepatic first-pass insulin clearance, leading to peripheral hyperinsulinemia. After TIPS, fasting insulin levels rise significantly, while C-peptide and proinsulin remain unchanged, supporting reduced hepatic insulin extraction rather than increased pancreatic secretion [116, 117]. Although glucose intolerance and glucagon levels may worsen, HbA1c and fasting glucose often remain stable during follow-up, suggesting no consistent clinically significant deterioration in glycemic control [118]. Conversely, occlusion of spontaneous portosystemic shunts, such as balloon-occluded retrograde transvenous obliteration, may improve insulin resistance and glucose tolerance by restoring portal venous flow and hepatic insulin clearance [119].

Prognostically, DM is associated with adverse post-TIPS outcomes, particularly HE, renal dysfunction, and shunt insufficiency. Large cohort studies identified DM as an independent predictor of overt and severe HE after TIPS [120, 121]. DM is also associated with early post-TIPS renal dysfunction, which correlates with higher mortality, while diabetes-related endothelial dysfunction and neointimal hyperplasia (bare stents or stent-grafts with incomplete coverage of the hepatic vein to the caval junction) may contribute to earlier shunt insufficiency [122, 123].

Importantly, not all diabetic patients carry the same risk. Adverse outcomes appear to be driven more by poor glycemic control and diabetes-related end-organ damage than by DM itself. Patients with diabetes and chronic complications have higher rates of mortality, renal complications, and HE, whereas diabetes without chronic complications may not confer the same excess risk [124].

Therefore, DM should be incorporated into individualized pre-TIPS risk assessment, especially for elective procedures. Optimization of glycemic control, evaluation of renal and cardiovascular complications, and careful post-TIPS monitoring are essential. Current prognostic models do not routinely include DM, but metabolic comorbidities may become increasingly relevant. In decompensated cirrhosis, insulin generally remains the preferred glucose-lowering therapy, while less stringent glycemic targets may be appropriate given the high risk of hypoglycemia [125].

### Obesity

Obesity is increasingly relevant in patients undergoing TIPS, particularly in MASH-related cirrhosis. However, body mass index (BMI) is unreliable in cirrhosis because it is confounded by ascites, edema, and altered body composition. CT-based assessment of visceral and subcutaneous adipose tissue, myosteatosis, and sarcopenic obesity provides more clinically informative measures.

Body fat distribution appears to influence post-TIPS outcomes, especially HE. A higher visceral-to-subcutaneous fat ratio has been associated with increased OHE risk and worse transplant-free survival after TIPS [126]. Conversely, low adipose tissue reserves may also increase HE risk in a sex-specific manner, with low visceral fat in men and low subcutaneous fat in women associated with higher post-TIPS HE risk [127]. Myosteatosis and subcutaneous adipose tissue indices may further improve HE prediction beyond skeletal muscle mass alone [128]. These findings suggest a complex, non-linear relationship between adiposity and post-TIPS complications, with fat distribution being more relevant than BMI-defined obesity.

Sarcopenic obesity, defined by reduced muscle mass with excess adiposity, is a high-risk phenotype associated with worse survival after TIPS and added prognostic value beyond model for end-stage liver disease (MELD) score, although it does not consistently predict post-TIPS HE [129].

TIPS may also modify body composition. After the procedure, subcutaneous fat tends to increase over time, likely reflecting improved nutritional status, while visceral fat may decrease early, possibly due to reduced portal pressure, improved portal drainage, and increased metabolic demands related to muscle anabolism [130]. These compartment-specific changes may therefore reflect both clinical improvement and metabolic remodeling.

In MASH-related cirrhosis, real-world data suggest that TIPS outcomes are broadly comparable to those in alcohol- or viral-related cirrhosis when patients are matched for disease severity and cardiometabolic comorbidities [131].

Thus, obesity or MASH etiology should not preclude TIPS when standard indications are present. Nevertheless, obese patients, particularly those with diabetes or metabolic syndrome, require careful pre-TIPS evaluation and post-procedural monitoring because of the metabolic effects of portosystemic shunting and the potential increased risk of HE.

### The gut microbiota

TIPS has bidirectional effects on gut microbiota and systemic inflammation. By reducing PH, it may partially improve cirrhosis-associated dysbiosis, gut barrier dysfunction, and bacterial translocation. Studies have reported increases in beneficial taxa, including short-chain fatty acid-producing bacteria, such as Faecalibacterium and Ruminococcaceae, together with reductions in pro-inflammatory metabolites and pathogenic bacterial families after TIPS [132, 133]. These changes may improve intestinal homeostasis and contribute to the gut–muscle–liver axis, potentially supporting muscle mass recovery in selected patients.

However, portosystemic shunting also allows gut-derived toxins and microbial metabolites to bypass hepatic clearance, increasing systemic exposure to endotoxins, ammonia, and neuroactive compounds. This mechanism is particularly relevant to post-TIPS HE. Although TIPS may acutely reduce hepatic endotoxin clearance, longer-term data suggest a net decrease in systemic inflammation, with reductions in soluble inflammatory markers, sCD14, IL-6, and CRP during follow-up [133, 134].

Ageing may further amplify this risk. Older patients often show reduced microbial diversity, loss of beneficial commensals such as Bifidobacteria, expansion of pathogenic taxa, delayed gut transit, altered bile acid metabolism, impaired mucosal immunity, and reduced intestinal barrier resilience [135–137]. In cirrhosis, these age-related changes may worsen bacterial translocation, endotoxemia, and chronic low-grade inflammation, reducing gut–liver axis resilience after portosystemic shunting [138]. Older adults may therefore be particularly vulnerable to post-TIPS HE, infections, renal dysfunction, and systemic inflammatory complications.

The microbiota profile differs between patients who develop post-TIPS HE and those who do not. HE has been associated with enrichment of potentially pathogenic or ammonia-related taxa, including Haemophilus, Eggerthella, Morganella, and Phocaeicola vulgatus, and depletion of beneficial butyrate-producing genera [139, 140]. Microbial metabolites may also contribute directly to neurotoxicity: increased phenylalanine decarboxylase genes, largely derived from Ruminococcus gnavus, promote phenylethylamine production, which has been linked to impaired cognition and higher post-TIPS HE risk [141]. Altered bile acid–microbiota signaling may further modulate HE severity [142].

These findings support a central role of the gut–liver–brain axis in post-TIPS HE, particularly in older adults with reduced physiological reserve. Pre-procedural assessment of nutritional status, frailty, sarcopenia, and microbiota-related risk factors may refine risk stratification. Microbiome-targeted interventions, including rifaximin, probiotics, prebiotics, synbiotics, dietary fiber, and fecal microbiota transplantation, are therefore of growing interest; however, evidence specifically in older patients undergoing TIPS remains limited and requires further validation [143–147].

## Addressing the unmet needs of older adults undergoing TIPS

**Figure 2:**
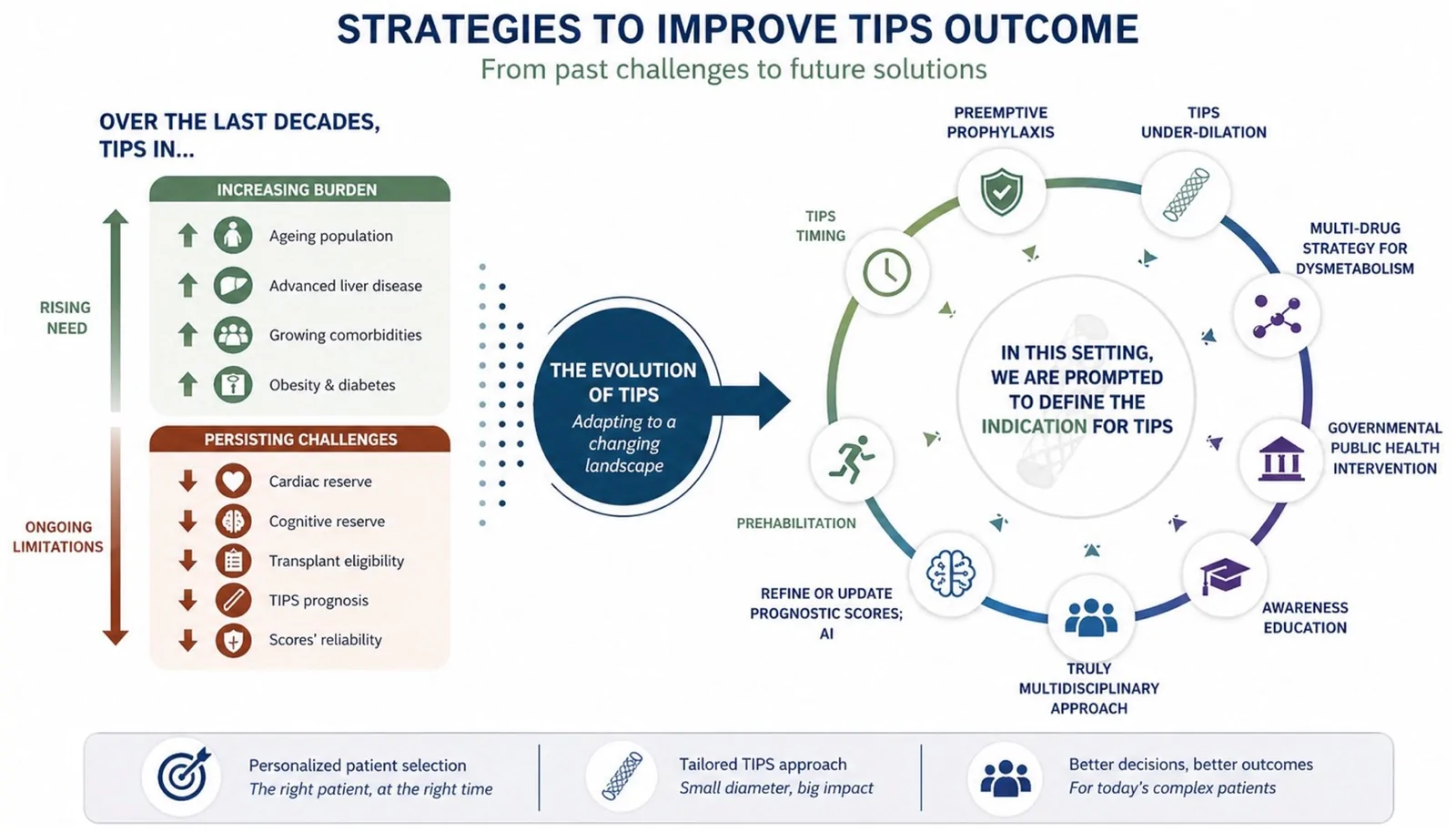
Over the past few decades, the profile of patients undergoing TIPS has undergone a profound transformation. Conditions marked by green upward arrows have progressively risen in prevalence, reshaping the overall clinical landscape. Conversely, determinants represented by maroon downward arrows have declined, collectively driving greater clinical complexity. Within this evolving scenario, accurately defining TIPS candidacy is crucial; the right panel outlines the key strategies proposed to optimize post-procedural outcomes. TIPS, transjugular intrahepatic portosystemic shunt.

Epidemiological trends indicate that an increasing proportion of patients develop severe manifestations of PH after the age of 70. Indeed, in both Western and Eastern regions, adults aged 65/70 years or older represent the fastest-growing subgroup of patients with cirrhosis, accounting for more than one-third of all advanced liver disease cases in some regions [12–14]. This demographic shift reflects improved survival in chronic liver disease, the rising burden of metabolically driven liver disease, and advances in the diagnosis and treatment of cirrhosis-related complications. In older adults, however, the indication for TIPS is increasingly complicated by the coexistence of age- and MASH-related comorbidities, particularly, although not exclusively, in the cardiovascular domain (Fig. 2).

As discussed in the previous sections, older adults referred for TIPS often present with reduced cardiovascular, hepatic, renal, metabolic, and neurocognitive reserve, together with a higher prevalence of obesity, frailty, sarcopenia, cognitive vulnerability, and alterations in the gut–liver axis. These features challenge conventional decision-making and call for a comprehensive reassessment of TIPS candidacy, procedural strategy, complication prevention, and post-procedural follow-up (Tables 2–4).

**Table 3:** Strategies to improve outcomes in older adults undergoing TIPS.^a^

Strategy	Description
**Careful patient selection**	Comprehensive pre-procedural assessment focusing on comorbidities (cardiac, renal, frailty status), functional reserve, and baseline risk of HE
**Frailty and geriatric assessment**	Use of frailty indices, sarcopenia evaluation, and multidimensional geriatric assessment to identify high-risk patients and support individualized decision-making.
**Optimized indication and timing**	TIPS should be reserved for well-established indications and ideally performed before advanced decompensation or severe organ dysfunction develops.
**Stent diameter modulation**	Use of smaller-diameter stent-grafts to reduce over-shunting and the risk of post-TIPS HE while preserving efficacy.
**Peri-procedural optimization**	Careful management of fluid status, renal function, and cardiac performance before and after the procedure to reduce complications.
**Prevention of hepatic encephalopathy**	Prophylactic lactulose and rifaximin, combined with early post-procedural monitoring, to reduce incidence and severity of HE.
**Close post-procedural follow-up**	Structured follow-up for early detection and management of complications such as HE, heart failure, and shunt dysfunction.
**Multidisciplinary management**	Collaboration among hepatologists, interventional radiologists, geriatricians, nutritionists, physiotherapists, nephrologists and cardiologists to improve risk stratification and outcomes.

a HE, hepatic encephalopathy; TIPS, transjugular intrahepatic portosystemic shunt.

**Table 4:** Domain-by-domain multidisciplinary framework for risk mitigation in older adults undergoing TIPS.^a^

Domain	Peri- and post-procedural mitigation
**Cardiovascular**	Small-diameter/under-dilated endoprosthesis; temporary withdrawal of non-selective beta-blockers; cautious diuretic titration; repeat right-heart catheterization immediately post-TIPS and at follow-up
**Renal**	Low-osmolar contrast with minimized load; avoid NSAIDs and other nephrotoxins; careful peri-procedural volume management; meticulous diuretic titration during ascites mobilization
**Neurocognitive (HE)**	Prophylactic lactulose; selective rifaximin; restrained shunt diameter; deprescribing of sedatives/opioids/anticholinergics; early shunt reduction for refractory HE
**Frailty and sarcopenia**	Prehabilitation–rehabilitation; nutritional support with adequate protein; physical therapy; staged goals of care
**Metabolic**	Optimize glycemic control and intravascular volume status; seek early endocrinology input; anticipate potential technical access difficulties.

a TIPS, trans-jugular intrahepatic portosystemic shunt; NSAIDs, non-steroidal anti-inflammatory drugs; HE, hepatic encephalopathy.

In this context, controlled under-dilation of TIPS endoprostheses has emerged over the past decade as a relevant technical refinement [148]. Initially developed to reduce over-shunting and post-TIPS complications, particularly HE, this strategy has shown clinical efficacy comparable to fully expanded stent-grafts in controlling PH-related complications, including events other than those that originally prompted TIPS [149–152] and irrespective of whether immediate post-TIPS hemodynamic targets recommended by current guidelines [3, 4] are achieved after TIPS.

Importantly, especially in patients with RA, under-dilated TIPS has also been associated with a survival advantage compared with TIPS dilated to diameters ≥8 mm, supporting the potential benefit of a more tailored approach to portal decompression [149–151].

These observations support an ongoing paradigm shift in the conceptual and technical approach to TIPS, with particular relevance for older adults [153]. Rather than pursuing maximal portal decompression, current strategies increasingly favor individualized portal pressure reduction. Indeed, lowering the portal pressure gradient below 10 mm Hg, or even normalizing it (<6 mm Hg), does not consistently translate into improved clinical outcomes. Conversely, excessive portosystemic shunting may increase the risk of HE, impairing quality of life, increasing caregiver burden and healthcare utilization, reducing functional independence, and, in severe or persistent cases, adversely affecting overall survival [154, 155]. Excessive shunting may also compromise liver function by reducing portal inflow and overwhelming the hepatic arterial buffer response, particularly in patients with advanced cirrhosis, older age, MASH-related liver disease, or concomitant cardiac dysfunction, in whom the capacity to increase compensatory hepatic arterial flow is often already diminished [156–159].

**Figure 3:**
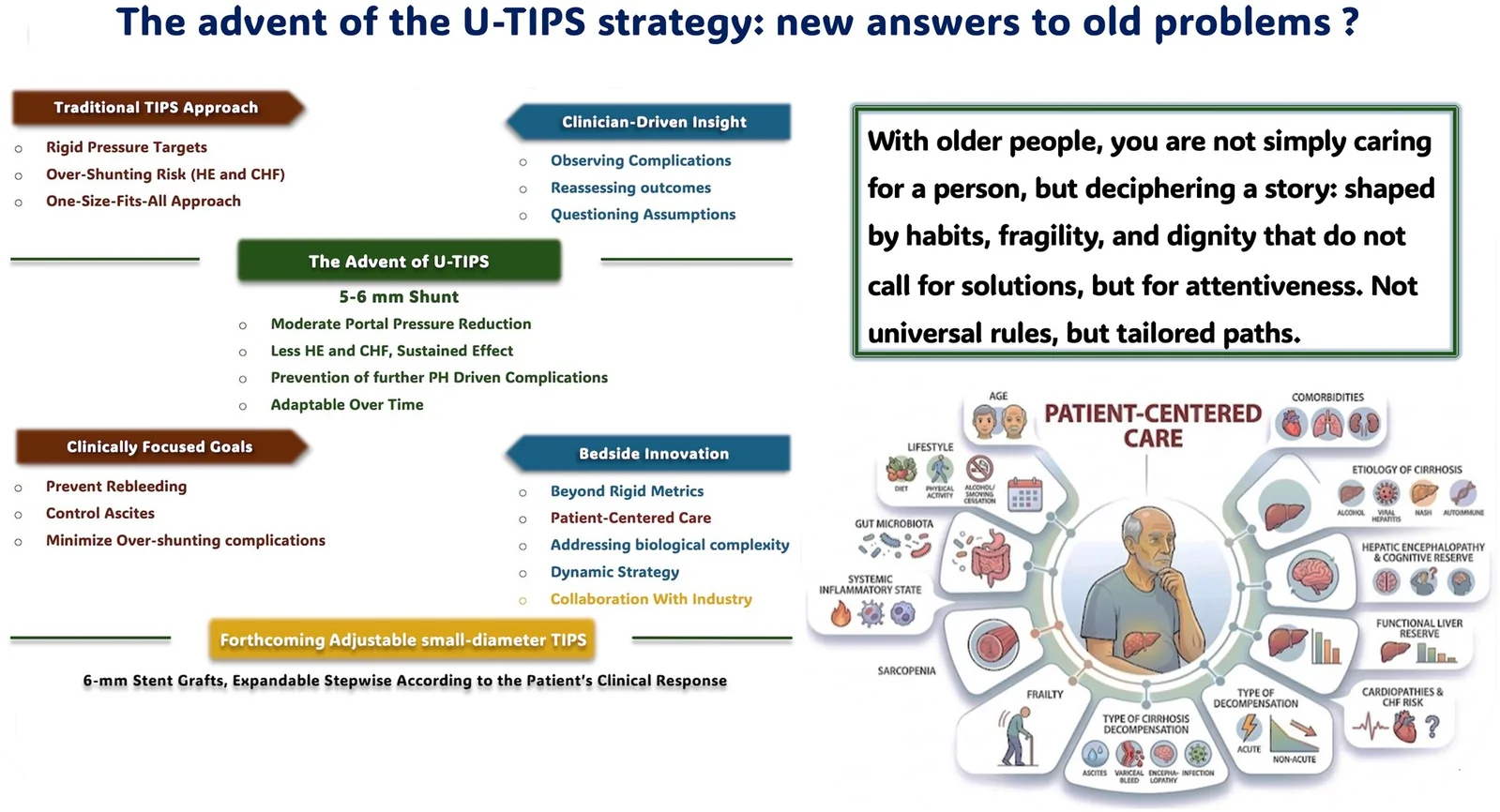
By moving away from a one-size-fits-all approach and the traditional reliance on standardized post-TIPS pressure targets, the under-dilated TIPS strategy reframes procedural choices around a more comprehensive, patient-centered assessment of outcomes. Such evolutionary shifts originate directly at the bedside, in clinics, wards, and interventional suites, where navigating the nuances of real-world patient complexity drives true medical innovation. CHF, congestive heart failure; HE, hepatic encephalopathy; PH, portal hypertension; U-TIPS, under-dilated transjugular intrahepatic portosystemic shunt.

Given the marked heterogeneity of TIPS candidates in terms of hepatic, cardiac, renal, and neurocognitive reserve, reliance on a single intra-procedural portal-caval pressure gradient target is unlikely to capture the complexity of individual risk. Accordingly, emerging evidence supports a transition from fixed pressure-based targets toward a dynamic, patient-centered strategy, in which post-TIPS clinical evolution guides the need for subsequent stepwise stent-graft dilation (Fig. 3) [153]. In this framework, portal pressure measurements should be regarded as surrogate markers rather than definitive predictors of outcome, as they cannot fully reflect the interaction between portal hemodynamics, systemic and cardiopulmonary circulation, hepatic functional reserve, and neurocognitive vulnerability (Fig. 1). While portal pressure reduction remains the cornerstone of TIPS efficacy, defining the threshold beyond which decompression becomes excessive remains a major unresolved challenge, thereby precluding a truly individualized approach to TIPS. Despite recent technical refinements, including the development of smaller diameter endoprostheses with an initial controlled intra-parenchymal caliber of 6 mm, optimal patient selection remains the key determinant of post-TIPS outcomes. This is particularly true in older adults, who often have reduced physiological reserve across multiple organ systems.

Currently available prognostic scores have limited discriminatory accuracy in this setting, largely because they were developed in epidemiological and clinical contexts that differ substantially from contemporary TIPS populations [22].

**Figure 4:**
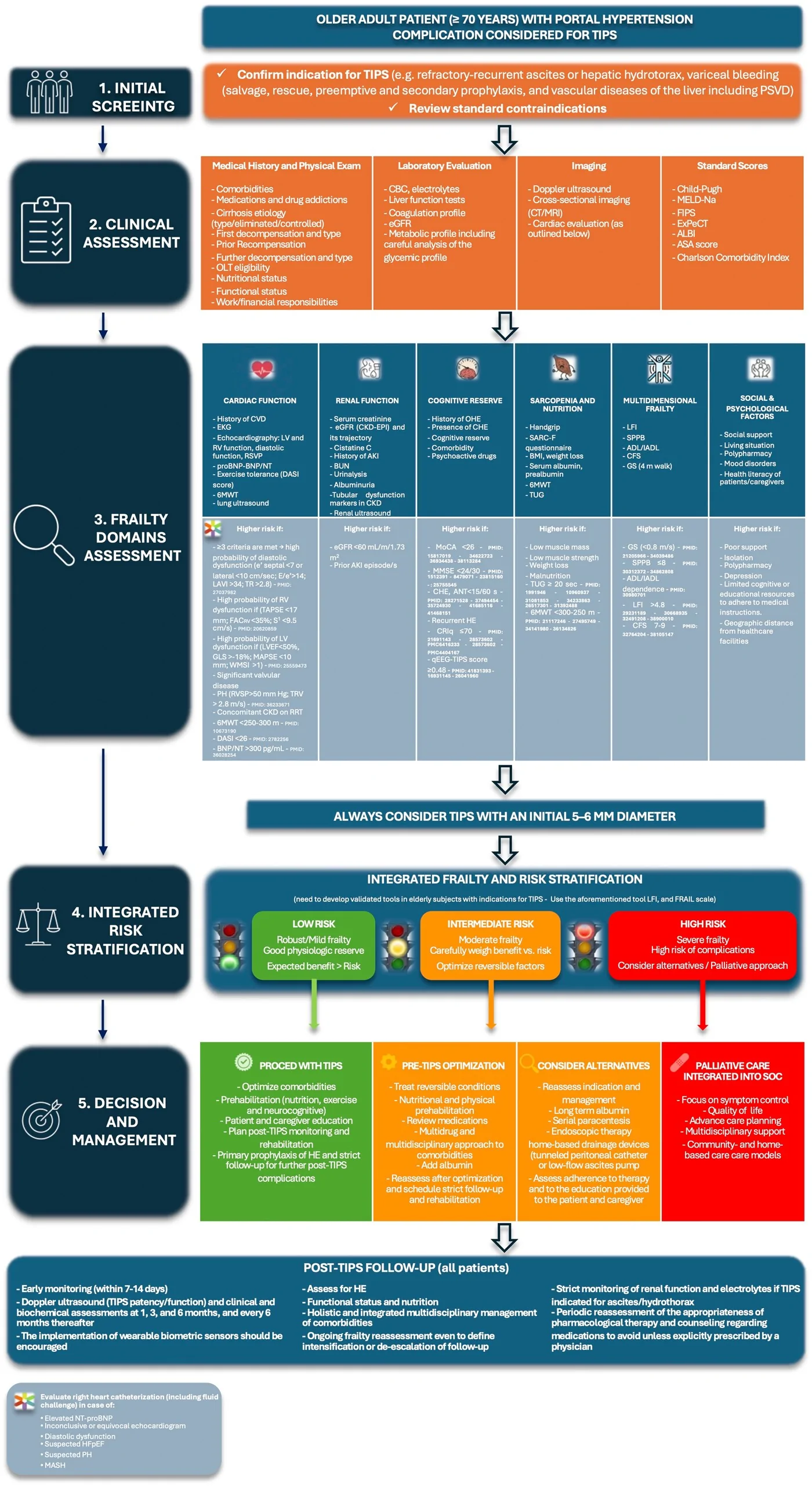
Although the literature does not support a specific global screening work-up, reliable parameters are available for each area of interest. These parameters should be integrated for the individual patient through a holistic assessment of older adults, defining their frailty in terms of comorbidities, transplant eligibility, pharmacological therapies, nutritional and functional status, as well as socio-economic and cultural context. When defining risk and identifying alternative strategies, it is essential to weigh the strength of the indication for TIPS, namely, the severity and prognostic impact of the PH complication. Therefore, the criteria listed in the table should not be viewed as dogmas, but rather as a guide to navigate an underlying complexity that cannot bypass clinical experience in managing both this patient population and the shunt procedure. The presence of frailty factors must guide operative choices, prompting technical optimization such as the use of small-diameter TIPS. Some of the tests listed may appear redundant; however, this reflects the variable local availability of the individual tools. The proposed list should therefore be understood as a selection of instruments considered to have the greatest potential diagnostic and prognostic impact. ADL, activity of daily living; AKI, acute kidney injury; ALBI, albumin–bilirubin score; ANT, animal naming test; ASA, american society of anesthesiologists physical status; BMI, body mass index; BNP, B-type natriuretic peptide; BUN, blood urea nitrogen; CBC, complete blood count; CFS, clinical frailty scale; CKD-EPI, chronic kidney disease epidemiology collaboration; CRIq, cognitive reserve index questionnaire; CVD, cardiovascular disease; EKG, electrocardiogram; DASI, Duke activity status index; EEG, electroencephalogram; eGFR, estimated glomerular filtration rate; ExPeCT, elderly patients calculator TIPS; FAC_RV_, fractional area change of the right ventricle; FIPS, Freiburg index of post-TIPS survival; GLS, global longitudinal strain; GSD, geriatric depression scale; GS, gait speed; HFpEF, heart failure with preserved ejection fraction; IADL, instrumental activity of daily living; LAVI, left atrial volume index; LFI, liver frailty index; LV, left ventricle; LVEF, left ventricular ejection fraction; MAPSE, mitral annular plane systolic excursion; MASH, metabolic dysfunction-associated steatohepatitis; MELD, model for end-stage liver disease; MMSE, mini-mental state examination; MoCA, montreal cognitive assessment; O- and C-HE, overt and covert hepatic encephalopathy; OLT, orthotopic liver transplant; PSVD, porto-sinusoidal vascular disorder; qEEG, quantitative electroencephalography; RRT, renal replacement therapy; RSVP, right ventricular systolic pressure; RV, right ventricle; SARC-F, strength-assistance with walking-rise from a chair-climb stairs-falls; SPPB, short physical performance battery; TAPSE, tricuspid annular plane systolic excursion; TIPS, transjugular intrahepatic portosystemic shunt; TRV, tricuspid regurgitation velocity; TUG, timed up and go test; SOC, standard of care; WMSI, wall motion score index; 6MWT, 6-minute walk test.

A comprehensive multidisciplinary approach is therefore required, integrating hepatology, interventional radiology, cardiology, geriatrics, nutrition, rehabilitation, and neurocognitive expertise (Fig. 4).

In older adults undergoing TIPS, neurological vulnerability represents an important unmet need in risk assessment, as age-related cognitive impairment, frailty, and HE may overlap clinically and share common determinants, with one potentially mimicking or masking the other. Mild pre-existing neurocognitive impairment may be difficult to distinguish from CHE, while post-TIPS cognitive decline may reflect either new or worsening, HE, progression of underlying neurodegenerative disease, or an interaction between these conditions. Therefore, neurological vulnerability should not be considered synonymous with HE, but rather as a multidimensional construct encompassing cognitive reserve, frailty, functional status, and susceptibility to HE. A structured pre-TIPS assessment followed by longitudinal evaluation may help distinguish pre-existing neurocognitive impairment from incident or worsening HE, thereby addressing an important unmet need in risk stratification of older patients undergoing TIPS.

Several additional unmet needs remain in routine clinical practice. Non-invasive tools are still insufficient to reliably assess procedural risk, particularly in patients undergoing TIPS for ascites, in whom evaluation of cardiovascular reserve remains challenging. Even invasive assessments, including right heart catheterization, may identify previously unrecognized contraindications but rarely predict the systemic hemodynamic adaptations that follow shunt creation. Future research should therefore focus on robust predictors of post-TIPS outcomes, including provocative testing strategies that better reproduce the hemodynamic perturbation induced by shunt opening, novel biomarkers, multiparametric risk scores, and artificial intelligence-supported models. Achieving this goal will require large, integrated datasets with standardized clinical phenotyping across the full spectrum of patients evaluated for TIPS, including but not limited to older adults.

Healthcare policy and organizational interventions are also needed to promote earlier identification of older adults who may benefit from portosystemic shunting, ensure closer longitudinal follow-up, support technological innovation, and refine the timing of TIPS across different indications. Timely intervention may be crucial: if TIPS is delayed until advanced stages of disease, the combined effects of progressive PH, hepatic dysfunction, and age- or disease-related frailty may outweigh the potential benefits of portal decompression. In such circumstances, TIPS may provide only limited symptomatic relief, with little or no meaningful effect on prognosis or residual quality of life and may even worsen both outcomes in selected patients.

Finally, structured post-TIPS follow-up is essential. Follow-up programs should actively involve trained caregivers able to recognize early shunt-related complications and ensure timely referral to specialist care. Emerging technologies, including wearable devices, may help detect early clinical deterioration and alert healthcare providers [160]. Post-TIPS care should also include nutritional counseling, prevention and early management of HE, neurocognitive monitoring, and physical rehabilitation or functional optimization. In elective candidates, these strategies may be further strengthened by prehabilitation before TIPS placement.

## Conclusions

Older adults constitute a rapidly expanding population of TIPS candidates and pose specific clinical challenges related to multimorbidity, metabolic dysfunction, frailty, cardiovascular limitations, renal vulnerability, and increased neurocognitive risk. Although outcomes are generally less favorable than in younger patients, chronological age alone should not be regarded as a contraindication to TIPS. Indeed, carefully selected older individuals may still derive meaningful survival and quality-of-life benefits from portal decompression.

Optimizing TIPS in this population requires a shift from conventional pressure-driven decision-making toward a multidimensional, patient-centered model of care. This should include careful assessment of comorbidities, frailty, functional reserve, cardiovascular and renal status, neurocognitive vulnerability, indication, and timing. Whenever feasible, TIPS should be considered before advanced decompensation and irreversible frailty limit its potential benefit. Technical refinements, including under-dilated or smaller-diameter stent-grafts, may further support individualized portal decompression by reducing the risk of over-shunting and post-TIPS HE while preserving clinical efficacy.

Peri-procedural optimization and structured follow-up are equally essential. Cardiac, renal, nutritional, and volume status should be carefully managed before and after TIPS, and preventive strategies for HE, including lactulose and rifaximin prophylaxis in selected patients, should be considered. Post-TIPS care should be delivered within a multidisciplinary framework involving hepatologists, interventional radiologists, geriatricians, cardiologists, nutrition specialists, rehabilitation teams, caregivers, and, when appropriate, palliative care services.

Beyond refining outcome assessment, future research should focus on strategies to prevent, promptly detect, and manage TIPS-related complications in older adults, particularly because liver transplantation is often not available as a rescue option in this population. Well-designed prospective studies are needed to define standardized screening pathways, validate frailty- and geriatric-informed risk models, clarify the optimal timing of TIPS across different indications, and assess the impact of small-diameter endoprostheses within individualized shunt-sizing strategies. Such evidence will be essential to guide decision-making between TIPS and alternative management approaches and to ensure that portal decompression is offered to older adults most likely to benefit from it.

## Authors’ contributions

D.R., D.S., and F.M was responsible for drafting and revising the manuscript; C.M, A.P., A.M, A.B., A.S.V.M, A.A., L.R., M.F., M.R., A.V., G.F., and F.F. performed the literature search and drafted the manuscript; S.M, F.S., and F.V. conceived the review and was responsible for drafting and revising the manuscript; all authors have read and approved the final version of the manuscript.
